# Analysis of the structure and function of the tomato Solanum lycopersicum L. MADS-box gene SlMADS5

**DOI:** 10.18699/VJ21.056

**Published:** 2021-09

**Authors:** A.V. Nezhdanova, M.A. Slugina, E.A. Dyachenko, A.M. Kamionskaya, E.Z. Kochieva, A.V. Shchennikova

**Affiliations:** Institute of Bioengineering, Federal Research Centre “Fundamentals of Biotechnology” of the Russian Academy of Sciences, Moscow, Russia; Institute of Bioengineering, Federal Research Centre “Fundamentals of Biotechnology” of the Russian Academy of Sciences, Moscow, Russia; Institute of Bioengineering, Federal Research Centre “Fundamentals of Biotechnology” of the Russian Academy of Sciences, Moscow, Russia; Institute of Bioengineering, Federal Research Centre “Fundamentals of Biotechnology” of the Russian Academy of Sciences, Moscow, Russia; Institute of Bioengineering, Federal Research Centre “Fundamentals of Biotechnology” of the Russian Academy of Sciences, Moscow, Russia; Institute of Bioengineering, Federal Research Centre “Fundamentals of Biotechnology” of the Russian Academy of Sciences, Moscow, Russia

**Keywords:** Solanum lycopersicum, Nicotiana tabacum, heterologous gene expression, MADS-domain transcription factors, SEPALLATA, SlMADS5, Solanum lycopersicum, Nicotiana tabacum, гетерологичная экспрессия гена, MADS-транскрипционные факторы, SEPALLATA, SlMADS5

## Abstract

At all stages of f lowering, a decisive role is played by the family of MADS-domain transcription factors, the combinatorial action of which is described by the ABCDE-model of f lower development. The current volume of data suggests a high conservatism of ABCDE genes in angiosperms. The E-proteins SEPALLATA are the central hub of the MADS-complexes, which determine the identity of the f loral organs. The only representative of the SEPALLATA3 clade in tomato Solanum lycopersicum L., SlMADS5, is involved in determining the identity of petals, stamens, and carpels; however, data on the functions of the gene are limited. The study was focused on the SlMADS5 functional characterization. Structural and phylogenetic analyses of SlMADS5 conf irmed its belonging to the SEP3 clade. An in silico expression analysis revealed the absence of gene transcripts in roots, leaves, and shoot apical meristem, and their presence in f lowers, fruits, and seeds at different stages of development. Two-hybrid analysis showed the ability of SlMADS5 to activate transcription of the target gene and interact with TAGL1. Transgenic plants Nicotiana tabacum L. with constitutive overexpression of SlMADS5 cDNA f lowered 2.2 times later than the control; plants formed thickened leaves, 2.5–3.0 times thicker stems, 1.5–2.7 times shortened internodes, and 1.9 times fewer f lowers and capsules than non-transgenic plants. The f lower structure did not differ from the control; however, the corolla petals changed color from light pink to magenta. Analysis of the expression of SlMADS5 and the tobacco genes NtLFY, NtAP1, NtWUS, NtAG, NtPLE, NtSEP1, NtSEP2, and NtSEP3 in leaves and apexes of transgenic and control plants showed that SlMADS5 mRNA is present only in tissues of transgenic lines. The other genes analyzed were highly expressed in the reproductive meristem of control plants. Gene transcripts were absent or were imperceptibly present in the leaves and vegetative apex of the control, as well as in the leaves and apexes of transgenic lines. The results obtained indicate the possible involvement of SlMADS5 in the regulation of f lower meristem development and the pathway of anthocyanin biosynthesis in petals.

## Abstract (ru)

На всех этапах цветения решающую роль играет семейство MADS-доменных транскрипцион- ных факторов, комбинаторное действие которых описывается ABCDE-моделью развития цветка. Современ- ный объем данных позволяет говорить о высоком консерватизме ABCDE-генов у покрытосеменных. Е-белки SEPALLATA являются центральным звеном MADS-комплексов, определяющих идентичность цветковых органов. Единственный представитель клады SEPALLATA3 томата Solanum lycopersicum L., SlMADS5, участвует в определении идентичности лепестков, тычинок и плодолистиков, однако данные о функциях гена ограни- чены. Целью работы стала функциональная характеристика SlMADS5. Структурно-филогенетический анализ SlMADS5 подтвердил его принадлежность к SEP3-кладе. Экспрессионный анализ in silico выявил отсутствие транскриптов гена в корнях, листьях и апикальной меристеме побега и их присутствие в цветках, плодах и се- менах на разных стадиях развития. Двугибридный анализ показал способность белка SlMADS5 активировать транскрипцию гена-мишени и взаимодействовать с белком TAGL1. Трансгенные растения Nicotiana tabacum L. с конститутивной сверхэкспрессией кДНК SlMADS5 цвели в 2.2 раза позже и формировали утолщенные ли- стья, имели в 2.5–3.0 раза более толстый стебель, в 1.5–2.7 раза укороченные междоузлия и в 1.9 раза меньше цветков и коробочек, чем нетрансгенные растения. Строение цветков от контроля не отличалось, однако ле- пестки венчика сменили окраску со светло-розовой на мадженту. Анализ экспрессии SlMADS5 и генов табака NtLFY, NtAP1, NtWUS, NtAG, NtPLE, NtSEP1, NtSEP2 и NtSEP3 в тканях листьев и апексов трансгенных и контроль- ных растений показал, что мРНК SlMADS5 присутствует только в тканях трансгенных линий. Остальные ана- лизируемые гены высоко экспрессировались в репродуктивной меристеме контрольных растений. Транс- крипты данных генов отсутствовали или присутствовали в следовых количествах в листьях и вегетативном апексе контроля, а также в листьях и апексах трансгенных линий. Полученные результаты свидетельствуют о возможном участии гена SlMADS5 в регуляции развития меристемы цветка и пути биосинтеза антоцианов в лепестках.

## Introduction

Throughout the plant’s life cycle, its root and shoot apical meristems maintain a pool of pluripotent stem cells, which give rise to new organs: roots and leaves respectively, during vegetative development and flowers during reproduction stage. At the reproductive stage, the shoot apical meristem of the angiosperms turns into the inflorescence meristem, which forms determined flower meristems (Hugouvieux et al., 2018). In all aspects of flowering, the MADS-domain family of transcription factors (TFs) plays a key role according to the well-known ABCDE flower development model (Smaczniak et al., 2012).

The ABCDE model is based on genetic and molecular studies, primarily of model species Arabidopsis thaliana (L.) Heynh., Antirrhinum majus L., and Petunia×hybrida hort. ex E. Vilm. (Coen, Meyerowitz, 1991; Angenent et al., 1995; Pelaz et al., 2000; Theissen, 2001; Ditta et al., 2004). According to the model, the identity of flower organs is determined by five classes of genetic activities: A and E – sepals; A, B and E – petals; B, C and E – stamens; C and E – carpels; C, E and D – ovules. At the molecular level, the ABCDE-model is explained by the so-called “quartet” model, according to which MADS-TFs of ABCDE classes in various combinations form tetramers: for example, C/C/E/E – to determine carpel identity, or A/B1/B2/E – to specify petal identity (Honma, Goto, 2001; Theissen, Saedler, 2001). These tetramers activate or suppress transcription of target genes (Melzer et al., 2009; Smaczniak et al., 2012). The current data suggest a high structural and functional conservatism of A, B, C, D, and E genes in flowering plants (Smaczniak et al., 2012).

The genes of the E-class, A. thaliana SEPALLATA (SEP1, SEP2, SEP3, and SEP4), which are involved in determining the identity of all floral organs, deserve special attention (Pelaz et al., 2000; Smaczniak et al., 2012). The knockout of only one of the SEP genes does not have a significant effect on the A. thaliana flower, while the sep1 sep2 sep3 triple mutation transforms all the flower organs into sepals; a new flower with the same development pattern is formed instead of the pistil (Pelaz et al., 2000). The quadruple sep1 sep2 sep3 sep4 mutation leads to the replacement of all flower organs with leaf-like organs (Ditta et al., 2004).

SEP proteins are the central hub in the formation of MADSTF quartets (Immink et al., 2009). Among SEPs, SEP3 is the most functionally pleiotropic and interacts with almost all MADS-TFs responsible for the identity of flower organs (Alhindi et al., 2017). SEP3 gene simultaneous ectopic expression expression with the A-, B-, or C-class genes transforms leaves into flower organs (Honma, Goto, 2001; Pelaz et al., 2001b).

During plant evolution, SEP genes are believed to have arisen later than other flower-related MADS-box genes, but at the same time they became key players in the origin of flowering plants, as well as in the domestication and breeding of crops (Theissen, 2001; Schilling et al., 2018). Therefore, their study in cultivated plants can expand the understanding of the role of these genes in determining economically valuable traits.

The tomato Solanum lycopersicum L. is one of the most important vegetables and, at the same time, a model for studying the fleshy fruit development and ripening. The tomato genome has been sequenced and annotated (https:// www.solgenomics.net/), and contains several SEP genes: TAGL2 (Solyc05g015750.2.1), SlMADS6/TM29/LeSEP1 (Solyc02g089200.2.1), RIPENING INHIBITOR ( MADSRIN) (Solyc05g012020.2.1), SlMADS98/SlCMB1 (Solyc04 g005320.2.1), SlMADS1/ENHANCER-OF-JOINTLESS-2 (Solyc03g114840.2.1), SlMBP21/JOINTLESS-2 (J2) (Solyc12g038510.1.1) and SlMADS5/TM5/TDR5/LeSEP3 (Solyc05g015750.3.1) (Wang Y. et al., 2019).

In addition to determining the flower organ identity, SEP proteins, together with MADS-TFs of the FRUITFULL (FUL) and AGAMOUS (AG) subfamilies, are actively involved in the regulation of fruit ripening. This is clearly demonstrated in tomato, the fruit ripening of which is controlled by FUL1/FUL2, TOMATO AGAMOUS 1 (TAG1)/TOMATO AGAMOUS-LIKE 1 (TAGL1) and MADS-RIN (Karlova et al., 2014; Shima et al., 2014; Wang R. et al., 2019). At the same time, FUL2 and TAGL1 have been shown to play an additional role in pistil initiation and early fruit development (Vrebalov et al., 2009; Wang R. et al., 2019), which is likely to be performed in combination with the tomato SEP3 homolog, SlMADS5 (Leseberg et al., 2008).

SEP1-like gene TAGL2 was shown to be expressed at stages I (anthesis) and II of the tomato fruit development (Busi et al., 2003). Suppression of SEP1-like TM29 causes the development of parthenocarpic fruits and the flower reversion (Ampomah-Dwamena et al., 2002). Tomato SEP4-like SlCMB1 regulates ethylene biosynthesis and the accumulation of carotenoids during fruit ripening; suppression of SlCMB1 leads to a change in the inflorescence architecture and an increase in the sepal size (Zhang et al., 2018a, b). SEP4-like SlMADS1 acts as a negative regulator of fruit ripening (Dong et al., 2013). SEP4-like SlMBP21 specifies the sepal size mediated by ethylene and auxin signaling, as well as the abscission zone formation (Li et al., 2017; Roldan et al., 2017). SEP4-like MADS-RIN is the main regulator of fruit ripening: gene knockout leads to the formation of an unripe fruit, including the absence of carotenoid accumulation (Vrebalov et al., 2002; Leseberg et al., 2008).

The only representative of the tomato clade SEP3, TF SlMADS5, is involved in determining the identity of the organs of the three inner flower whorls (Pnueli et al., 1994), interacting with MADS-TFs of the SEP and AG subfamilies (Leseberg et al., 2008). Despite the SEP3 significance, this gene variability has been characterized in cultivated and wild tomato species, and the SlMADS5 expression was observed in some organs and tissues (Pnueli et al., 1994; Slugina et al., 2020).

The aim of the present study was to characterize the function of S. lycopersicum SlMADS5. SlMADS5 structural, phylogenetic and expression analysis confirmed its belonging to the SEP3-clade. Analysis in the yeast two-hybrid GAL4-system showed the SlMADS5 TF activator properties and its interaction with C-class MADS-TF. Transgenic Nicotiana tabacum L. plants with SlMADS5 constitutive overexpression exhibited a pronounced phenotype of reproductive development suppression.

## Materials and methods

Tomato S. lycopersicum cv. Silvestre recordo and tobacco N. tabacum cv. Samsun plants were used in the study. Tomato accessions were grown under controlled greenhouse conditions (day/night: +21/23 °C, 16 h/8 h; 300–400 μmol/m–2/s–1) until flowering. Roots, leaves, flowers and ripe fruits were collected separately. Tissues were grounded in liquid nitrogen and stored at –70 °C. Tobacco accessions were grown in vitro on a sterile MS medium in a climatic chamber (day/night: +21/23 °C, 16 h/8 h; 300 μmol/m–2/s–1) until the formation of 4–6 leaves.

Total RNA was isolated from tomato (roots, leaves, flowers, and ripe fruits) and tobacco (leaves, vegetative apex, and reproductive apex) tissues using the RNeasy Plant Mini Kit (QIAGEN, USA), and used for cDNA synthesis (the Reverse Transcription System, Promega, USA). Genomic DNA was isolated from leaf tissues by the standard potassium acetate method (Dyachenko et al., 2018) and used for PCR tests for the presence of a transgene in the plant genome.

Primers for gene amplification, sequencing, and expression analysis were generated based on the MADS-box transcripts of S. lycopersicum cv. Heinz and tobacco N. tabacum genes available in the NCBI (http://www.ncbi.nlm.nih.gov/) (NtAPETALA1 (NtAP1; JQ686939.1, AF068724.2, XM_016635359.1, AF009127.1, U63162.1); NtLEAFY (NtLFY; JQ686928.1, XM_016593842.1); NtWUSCHEL (NtWUS; XM_016637596.1, MG843891.1, XM_016619508.1, JQ686923.1); NtAG (NM_001325900, XM_016638054.1, XM_016580096.1, XM_016580095.1, XM_016580097.1); NtPLENA (NtPLE; XM_016631079.1, XM_016631071.1, XM_016615571.1, XM_016615578.1, U63163.1): NtSEP1 (XM_016653813.1, XM_016645589.1, XM_016620650.1, XM_016596552.1, XM_016611481.1, XM_016645132.1, NM_001324748.1, XM_016620651.1, XM_016647424.1, XM_016644825.1); NtSEP3 (NM_001325160.1, XM_016582910.1); NtSEP2 (XM_016645132.1, NM_001324748.1, XM_016645589.1)) so that forward and reverse primers are separated by at least one intron and match all possible transcripts for each of the analyzed genes (Table 1). The primer sequences were additionally verified using Primer 3 and BLAST (https://www.ncbi.nlm.nih.gov/ tools/primer-blast/). Primers for CDS in-frame cloning into plasmid vectors (GAL4 system) contained EcoRI (forward, F) and SalI (reverse, R) restriction sites at the 5′ end.

**Table 1.**
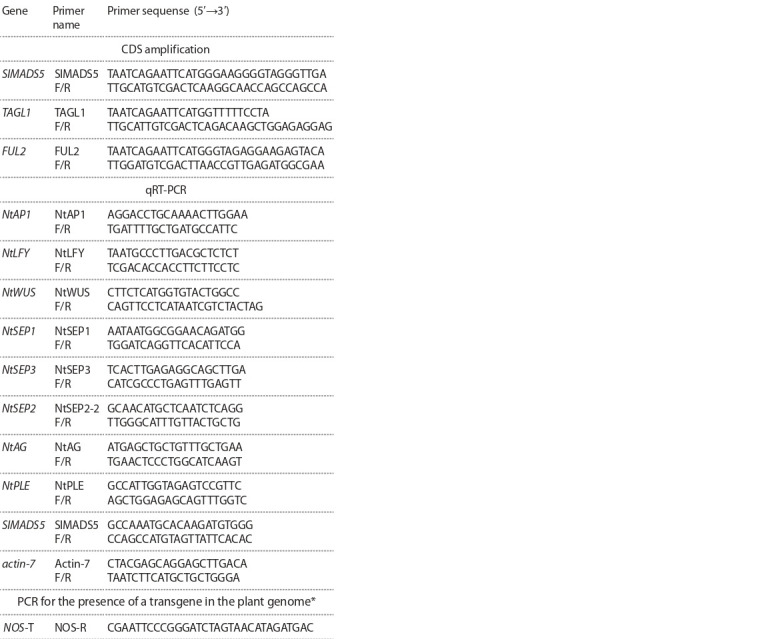
The list of primers used in the study * SlMADS5-F primer was used as a direct primer for PCR analysis of plants for the presence of a transgene in the genome.

Full-length SlMADS5, TAG1, and FUL2 cDNAs were amplified using the cDNA, isolated from S. lycopersicum cv. Silvestre recordo flowers; PCR conditions: initial denaturation at 95 °C for 5 min; 30 cycles of denaturation (94 °C for 30 s), annealing (55 °C – 30 s) and synthesis (72 °C – 1 min); final synthesis (72 °C – 7 min). The PCR fragments of the expected length were purified using the MinElute Gel Extraction Kit (QIAGEN, USA), cloned into the pGEM®-T Easy plasmid vector (Promega, Madison, WI, USA) at EcoRI and Sal I sites and sequenced (Core Facility “Bioengineering”). Further, the SlMADS5, FUL2, and TAGL1 CDSs were cloned into hybrid vectors pAD-GAL4 and pBD-GAL4cam (Aglient Technologies, USA): each gene was ligated in frame with the activator domain (pAD) and DNA-binding domain (pBD) of the yeast TF GAL4. Recombinant pJ69-4a strains carrying each pADgene and pBD-gene construct separately, as well as in pairs pAD-gene + pBD-gene, were obtained. For plant transformation, SlMADS5 cDNA was cloned in a sense orientation into a binary vector based on pBin19, under the control of the enhanced cauliflower mosaic virus promoter 35S and nopaline synthase (NOS) terminator. With this construct, a recombinant agrobacterial strain AGLØ was obtained.

For sequence structural analysis, the NCBI-CDD (http:// www.ncbi.nlm.nih.gov/Structure/cdd/wrpsb.cgi), MEGA 7.0 (Kumar et al., 2016) and Phyre2 (http://www.sbg.bio.ic.ac. uk/phyre2/) were used. Sequence phylogeny was assessed in the MEGA7, using Maximum Likelihood method based on the JTT model.

Gene expression analysis was performed in silico (using TomExpress database; http://tomexpress.toulouse.inra.fr/ select-data), as well as by quantitative (q) real-time (RT) PCR in two biological and three technical replicates. The kit “Reaction mixture for carrying out qRT-PCR in the presence of SYBR Green I and ROX” (JSC Syntol, RF) and the CFX96 Real-Time PCR Detection System (Bio-Rad Laboratories, USA) were applied. The qRT-PCR conditions were as follows: 95 °C – 5 min; 40 cycles (95 °C – 15 s, 60 °C – 50 s). The reference gene actin-7 (XM_016658880.1) (Schmidt, Delaney, 2010) was used for normalizing the expression of tobacco genes. Statistical processing of the results was carried out using the GraphPad Prism v. 7.02 (https://www. graphpad.com).

The analysis of SlMADS5 interactions with TAGL1 and FUL2 proteins was carried out in vivo in a two-hybrid GAL4- yeast system using the Saccharomyces cerevisiae Pj69-4a strain, according to the HybriZAP-2.1-Hybrid cDNA Two- Hybrid Synthesis Kit protocol (Stratagene).

Leaf explants of tobacco (N. tabacum cv. Samsun) were transformed using Agrobacterium tumefaciens strain AGLØ. To select transgenic regenerants, an MS medium containing kanamycin (Km, 100 mg/L) for selection and carbenicillin (500 mg/L), which suppresses agrobacteria growth, was used. The rooted regenerants were adapted to the soil in greenhouse conditions and then tested for the presence of a transgene in the genome by PCR with primers specific to the sequences of the 5′ end of the transgene and the NOS-terminator (see Table 1).

## Results

To confirm the conservatism of the SlMADS5 function in tomato (cv. Silvestre recordo), an analysis of its interactions with MADS-TFs TAGL1 and FUL2, the interaction with which was and was not, respectively, shown earlier (Leseberg et al., 2008), was carried out.

**Fig. 1.**
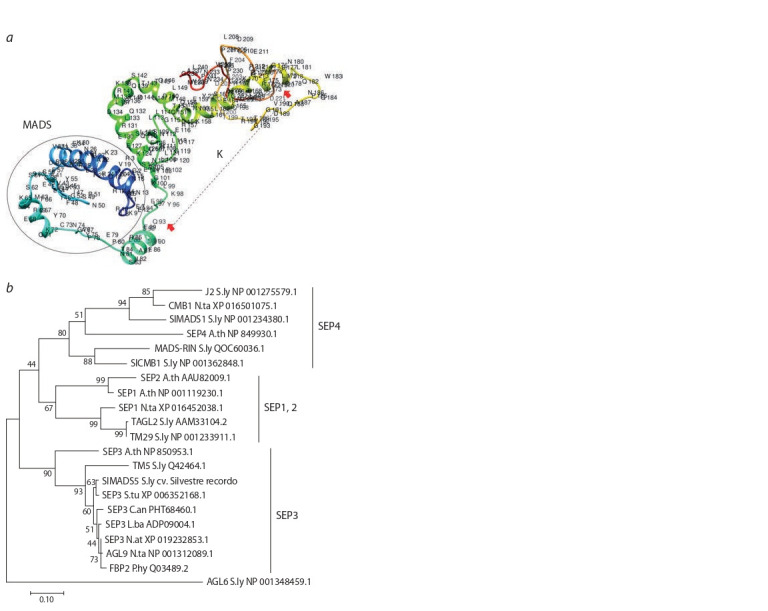
Structure and phylogenetic analysis of the SlMADS5. a – three-dimensional structure of TF SlMADS5 according to Phyre2. The MADS-domain is indicated with a circle; the beginning and end of the K-domain are indicated with red arrows; b – dendrogram based on the alignment of 19 MADS-TF sequences from the SEP clade of tomato, other Solanaceae species, and the model species Arabidopsis thaliana. The analysis was carried out in MEGA 7.0, using the Maximum Likelihood method based on the JTT model. The tree is rooted with S. lycopersicum AGL6 homolog. The signif icant bootstrap values for 1000 replicates are shown at the base of the branches. The NCBI accession numbers are shown opposite the protein names. S.ly – S. lycopersicum, N.ta – N. tabacum, A.th – A. thaliana, S.tu – S. tuberosum L., C.an – Capsicum annuum L., L.ba – Lycium barbarum L., N.at – N. attenuate Torr. ex S. Watson, P.hy – Petunia × hybrida.

Structural analysis of the SlMADS5 protein was carried out in comparison with the known tomato, tobacco, and A. thaliana SEP homologs. The presence of the main domains characteristic of MIKCc type MADS-TFs was confirmed, namely the highly conserved MEF2-like MADS-domain (1–76 aa), an I-region (77–92 aa), a conserved keratin (K)-like domain (93–173 aa), and a variable C-region (174–241 aa) (Fig. 1, a). The performed phylogenetic analysis testified the belonging of SlMADS5 to the SEP3 clade (see Fig. 1, b).

To characterize TF SlMADS5 functionally, we analyzed the expression of the SlMADS5 gene in various tomato organs and the ability of SlMADS5 protein to activate gene transcription and interact with MADS proteins of the C and A classes. Also, transgenic N. tabacum model plants with constitutive overexpression of SlMADS5 cDNA were obtained.

**Fig. 2.**
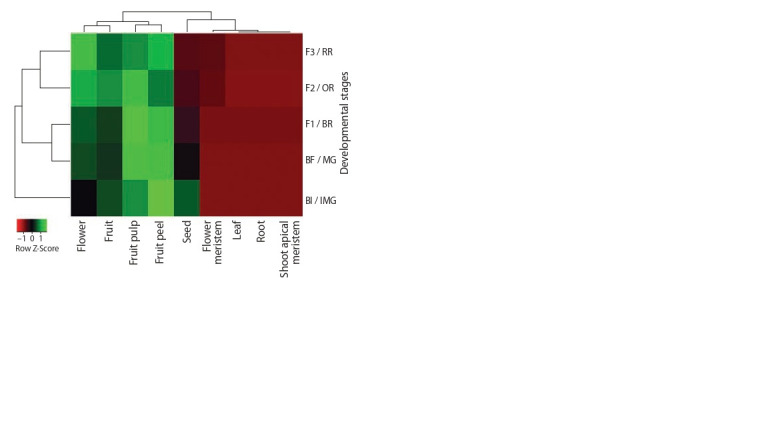
Heatmap of SlMADS5 gene expression in roots, shoot apical meristems, leaves, f lower meristems, and f lowers at the stages of bud initiation (BI), bud formation (BF), f lower opening (F1–F3), as well as in whole fruits, fruit peels, fruit pulps, and seeds at the stages IMG, MG, BR, OR, and RR. Expression of SlMADS5 in roots and reproductive tissues is shown for S. lycopersicum cv. MicroTom; in leaves and shoot apical meristems, for cv. M82.

In silico analysis of the SlMADS5 expression pattern was carried out in roots, leaves, vegetative shoot meristem, flower meristem, flower (from bud to fully open and anthesis stage), fruits (4–8 days after anthesis), fruit skin and pulp (stages: Immature Green (IMG); Mature Green (MG); Breaker (BR), color change; Orange (OR); Red Ripe (RR)), and in seeds (IMG, MG, BR, RR) (Fig. 2). SlMADS5 transcripts were not found in roots, leaves, and the vegetative apical meristem. At the same time, SlMADS5 expression was shown in flowers (maximum – at the anthesis stage), fruits, fruit peel (maximum at MG and BR stages), fruit pulp (maximum at IMG, MG, and BR stages), and seeds (maximum at IMG stage) (see Fig. 2).

In vivo analysis in the yeast two-hybrid GAL4 system showed that TF SlMADS5 has the property of activating the transcription of target genes, interacts with the C-class MADS protein TAGL1, but does not interact with the A-class MADS protein FUL2 (Table 2).

**Table 2.**
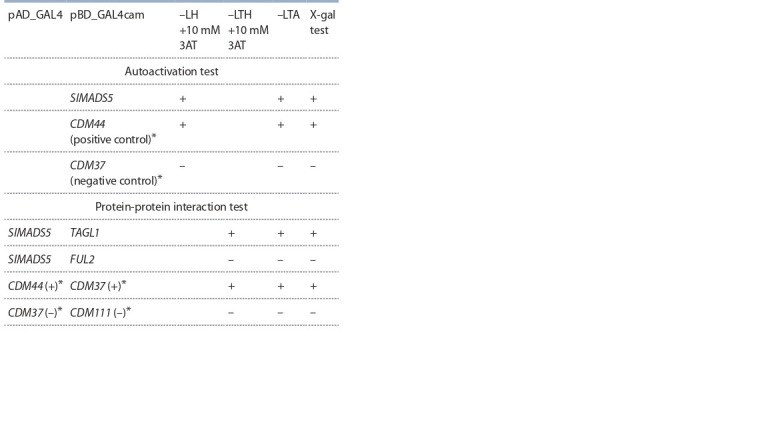
Results of the analysis of SlMADS5 protein-protein interactions * According to (Shchennikova et al., 2004). The experiment was carried out in parallel at room temperature and 30 °C (the same results were obtained for both temperatures). L – L-Leucine; H – L-Histidine; T – L-Tryptophan; A – L-Adenine hemisulfate salt; 3AT – 3-amino-1,2,4-triazole; –LH, –LTH and –LTA – nutritional medium without leucine/histidine, leucine/histidine/tryptophan, and leucine/ tryptophan/adenine, respectively; X-gal – 5-bromo-4-chloro-3-indolyl-β-D-ga-lactopyranoside. X-gal test: yeast colonies, where the analyzed proteins interact and, as a result, activate the expression of the β-galactosidase (lacZ) gene, acquire a blue color due to the cleavage of the X-gal substrate added to the medium by the β-galactosidase enzyme.

**Fig. 3.**
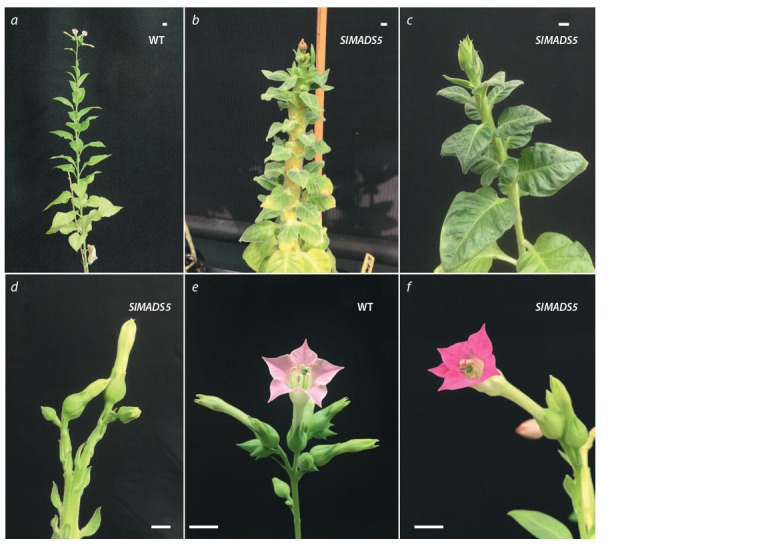
Transgenic tobacco plants Т0 (SlMADS5) (b–d, f ) in comparison with the control non-transgenic N. tabacum plant (WT) (a, e) at the stages of bud formation (c, d ), f lowering (a, e, f ), and seed formation (b). (c) and (d) – the top of the same plant 35S::SlMADS5. The photos were taken one and a half weeks apart. Scale bar 1 cm.

The characterization of transgenic tobacco plants with SlMADS5 constitutive overexpression was performed. Independent regenerants T0 35S::SlMADS5 (18 plants) were adapted to the greenhouse, tested by PCR for the presence of a transgene expression cassette in the genome, and compared with the control (non-transgenic tobacco plants) during development. In comparison with the control, 35S::SlMADS5 plants (Fig. 3) bloomed much later (on average, 138 days vs. 62 in the control). Also, 35S::SlMADS5 phenotype was characterized by a 2.5–3.0 times thicker stem, 2.0 times shortened internodes, thickened and darker leaves, and 2.5 times fewer flowers and capsules. The 35S::SlMADS5 flower structure did not differ from the control.

Seeds of two transgenic T0 lines (S5-16 and S5-17) with a pronounced phenotype were planted in a greenhouse. T1 plants, which gave a positive PCR signal for the presence of a transgene in the genome, bloomed 1.3–1.5 times later than the control, had a 35S::SlMADS5 phenotype, and formed flowers with magenta-colored corolla petals, in contrast to light pink petals in the control.

Seeds of lines T1 S5-16-6, S5-16-7, S5-17-1 and S5-17-4 were planted on MS medium (Km 50 mg/l); the 3:1 ratio of the number of Km-resistant to Km-sensitive seedlings indicated a heterozygous state of the transgene and one copy of it in the genome of transgenic lines. In seedlings, internodes were near absent, and only T2 plants of the S5-16-7 line (14 accessions) formed a noticeable stem and were adapted to the greenhouse (the rest of the plants died after transfer to the soil). Plants T2 S5-16-7 demonstrated the 35S::SlMADS5 phenotype: they bloomed 2.4 times later than the control; formed thickened stems and leaves, shortened internodes, and 2.3 times less seed capsules.

In T1 lines S5-16-7 and S5-17-1, in comparison with the control, we analyzed the SlMADS5 expression, as well as the expression of tobacco genes associated with reproductive development: NtLFY, NtAP1 (plant transition to flowering), NtWUS (central regulator of stem cells in the meristem), NtAG, NtPLE, NtSEP1, NtSEP2, NtSEP3 (key genes for the identity of the flower meristem and flower organs). For the analysis, we used tissues of leaves and apical meristems (vegetative and reproductive in the control, and shoot meristem in lines S5-16-7 and S5-17-1) of transgenic and control plants.

**Fig. 4.**
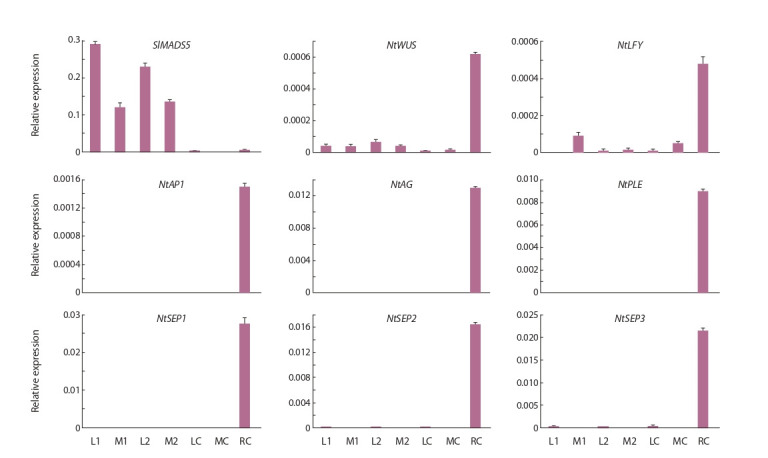
Expression of SlMADS5 and N. tabacum genes NtWUS, NtLFY, NtAP1, NtAG, NtPLE, NtSEP1, NtSEP2 and NtSEP3 in control (C) and transgenic lines S5-16-7 (1) and S5-17-1 (2). L – leaf; M – shoot apical meristem, R – reproductive meristem.

Expression of the SlMADS5 transgene was present only in the tissues of S5-16-7 and S5-17-1 plants. The expression pattern of the remaining analyzed genes was similar: their mRNA was absent or was minimal in the leaves of the control and transgenic lines, as well as in the S5-16-7 and S5-17-1 apexes of undefined status. At the same time, these genes were highly transcribed in the reproductive meristems of control plants (Fig. 4).

## Discussion

In this study, a functional analysis of the SlMADS5 gene, the SEP3 homolog in tomato, was carried out. Structural analysis (see Fig. 1) confirmed that SlMADS5 belongs to the SEP3 clade, which may indicate the conservatism of its role in the reproductive development of tomato, namely, its participation in determining the identity of petals, stamens, carpels, and ovules.

It is known that SlMADS5 is not expressed in tomato leaves and roots and is expressed in flowers and fruits (Slugina et al., 2020). Also, SlMADS5 mRNA is present in the meristem domains that correspond to the future three inner whorls of the tomato flower, as well as during organogenesis and in the corresponding mature organs (Pnueli et al., 1991, 1994). A detailed in silico analysis of the SlMADS5 expression pattern carried out in this study revealed that SlMADS5 mRNA is absent not only in roots and leaves, but also in the shoot apical meristems and flower meristems at early stages of development (see Fig. 2). Gene transcription is activated late in the development of the flower meristem, and reaches a peak in an open flower and in the peel of an immature fruit (see Fig. 2). This corresponds not only to the well-known role of SEP3 homologs in determining the differentiation of flower meristem cells corresponding to the three inner whorls of organs (Pnueli et al., 1991, 1994), but also suggests the active participation of SlMADS5 in the aspects of development and ripening of tomato fruits and seeds.

To characterize the SlMADS5 function, transgenic tobacco plants with constitutive overexpression of SlMADS5 cDNA were obtained. The phenotype of transgene overexpression does not determine its function; however, it may indicate a similarity with the already characterized homologs. Earlier, the effect of heterologous overexpression of SEP3 homologs of different plant species was studied mainly using transgenic A. thaliana plants, but there are works with the use of Nicotiana spp. plants. Tobacco, like tomato, belongs to the Solanaceae family and has the same flower structure; therefore, in this study, a heterologous expression system in tobacco was selected.

Various effects of overexpression of SEP3 homologs have been described. Thus, SEP3 constitutive expression in A. thaliana significantly accelerates flowering (Pelaz et al., 2001a). In these plants, the APETALA3 (B-class) and AG (C-class) genes are transcribed ectopically (Castillejo et al., 2005). Overexpression of the P. × hybrida SEP3-like gene FBP2 leads to early flowering of the A. thaliana plants (Ferrario et al., 2003). Early flowering is caused by overexpression of tobacco SEP3-like gene NsMADS3 in N. sylvestris Speg. & Comes (Jang et al., 1999) and chrysanthemum SEP3-like gene CDM44 in N. tabacum (Goloveshkina et al., 2012).

At the same time, no influence of overexpression of SEP3- homologous genes on the flowering time was also observed. Thus, homologous overexpression of FBP2 in P.×hybrida has no effect on plant vegetation period (Ferrario et al., 2006). Heterologous overexpression of Platanus acerifolia SEP3-like genes in A. thaliana causes early flowering only in the case of the PlacSEP3.2 gene, while overexpression of the second gene, PlacSEP3.1, causes early flowering only in transgenic tobacco plants (Zhang et al., 2017).

In the case of SlMADS5 constitutive overexpression, a significant delay in flowering was observed, most likely associated with the incorrect development of the shoot apical meristem (see Fig. 3). Different effects of heterologous ectopic expression of SEP3 homologs in transgenic plants may be associated with structural differences in encoded protein sequences responsible for binding to promoters of target genes or to partner proteins.

Normally, traces of the A. thaliana SEP3 transcripts are found in the inflorescence meristem, and gene expression is noticeably activated only in the flower meristem parts, from which petals, stamens, and carpels are subsequently formed (Ferrario et al., 2003; Urbanus et al., 2009). Therefore, the presence of the TF SlMADS5 in tissues, where there should be no tobacco SEP3 homologs, can lead to nonspecific proteinprotein and DNA-binding interactions of SlMADS5, which can disrupt the pattern of meristem development.

To clarify the status of transgenic meristems S5-16-7 and S5-17-1, visually ready for flowering, we analyzed the expression of genes whose activity is associated with the identity of the reproductive inflorescence and flower meristems (NtLFY and NtAP1) (Weigel et al., 1992). Considering the results 

obtained (see Fig. 4), only the inflorescence meristem of the control plant has reproductive status. The presence of a low level of LFY expression in the vegetative apex of the control and in the S5-16-7 apex (see Fig. 4) suggests the initial stages of the meristem transition to the reproductive state, since it has been shown that in A. thaliana LFY begins to be expressed in the flower meristem primordia at the periphery of the inflorescence meristem (Weigel et al., 1992).

It is known that SEP3 is the central hub of the MADScomplexes in A. thaliana (Immink et al., 2009). TF SlMADS5 also shows an exceptional ability to assemble tetrameric complexes of MADS TFs (Leseberg et al., 2008). The interaction of SlMADS5 with FUL2 and TAGL1 shown in this work (see Table 2), as well as the role of FUL2 and TAGL1 in pistil initiation and early fruit development (Vrebalov et al., 2009; Wang R. et al., 2019), indicate the possible involvement of SlMADS5 in determining the identity of the tomato pistil in complex with FUL2 and TAGL1.

One of the complexes, SEP3/SEP3/AG/AG, is required for flower determination and completion of its development (Hugouvieux et al., 2018). This is due to a decrease in the number of stem cells because of the WUS gene suppression with the key participation of TF AG (Lenhard et al., 2001). Accordingly, in transgenic petunia plants with simultaneous overexpression of SEP3-like FBP2 and D-class gene FBP11, where developmental arrest is observed at the cotyledon stage, transcription of AG-like FBP6 is activated and mRNA of WUS-like TERMINATOR is absent (Ferrario et al., 2006). This suggests the joint participation of SEP3, AG, and D-class genes in the suppression of stem cells in the meristem.

Taking into account the activation of AG expression in A. thaliana with SEP3 overexpression (Castillejo et al., 2005), as well as the participation of SEP3 and AG in the suppression of WUS transcription (Lenhard et al., 2001; Ferrario et al., 2006) and the interaction of TF SlMADS5 with the AG homolog TAGL1 (see Table 2), it can be assumed that the ectopically synthesized TF SlMADS5 is able to activate transcription of the tobacco AG-like genes NtAG and NtPLE in transgenic shoot meristem. Subsequent formation of complexes SlMADS5/SlMADS5/NtAG/NtAG or SlMADS5/ SlMADS5/NtPLE/NtPLE can lead to inhibition of meristem development due to the tobacco WUS-like gene NtWUS suppression, since WUS plays a key role in determining the stem cell identity, the population of which is not supported in plants with loss of WUS function (Ferrario et al., 2006; Jha et al., 2020).

To test this possibility, we analyzed the expression of SlMADS5, NtWUS, AG-like genes NtAG and NtPLE, as well as SEP-like genes NtSEP1, NtSEP2, and NtSEP3. However, the presence of SlMADS5 ectopic expression did not lead to the activation of AG-like genes, and the expression of NtWUS was significantly higher in the tissues of transgenic lines in comparison with the control (excluding the control inflorescence meristem) (see Fig. 4). The latter can be a probable reason for the formation of significantly thickened, in comparison with the control, stem and leaves of transgenic plants of all 11 lines with the 35S::SlMADS5 phenotype (see Fig. 3) as a result of the increased number of stem cells and the meristem overgrowth.

It should also be noted that in transgenic plants, the anthocyanin color of the flower corolla changed from pale pink (control) to magenta (35S::SlMADS5) (see Fig. 3). Previously, it was shown that the expression of the SEP-like gene MrMADS01 in Myrica rubra berries significantly increases at the last stage of ripening, which allowed the authors to suggest the involvement of this gene in the biosynthesis of anthocyanins (Zhao et al., 2019). Silencing the SEP-like gene PaMADS7 in sweet cherry (Prunus avium) leads to a change in the content of anthocyanins in fruits (Qi et al., 2020). It can be assumed that SlMADS5 is also involved in the regulation of anthocyanin biosynthesis in transgenic tobacco petals.

Silencing of SlMADS5 gene leads to a change in the number of flower whorls and the number of organs in whorls, as well as the formation of green petals with signs of sepals, and sterile anthers and carpels with signs of sepals and petals, respectively (Pnueli et al., 1994), which may indicate the participation of the gene in determining the identity of tomato flower organs. Nevertheless, no complete homeotic transformation of certain flower organs was observed when SlMADS5 was suppressed (Pnueli et al., 1994).

## Conclusion

The data on the effect of SlMADS5 overexpression on the development of transgenic tobacco plants obtained in this study also do not confirm the involvement of the gene in determining the floral organ identity. Also, the data obtained may indicate that the ectopic expression of this single gene in a heterologous system (N. tabacum) is insufficient to activate transcription of the MADS-box tobacco genes associated with flowering, but it is sufficient for a long delay in the reproductive development of the plant.

## Conflict of interest

The authors declare no conflict of interest.
